# Extrusion-Based 3D Printing of Rutin Using Aqueous Polyethylene Oxide Gel Inks

**DOI:** 10.3390/pharmaceutics17070878

**Published:** 2025-07-03

**Authors:** Oleh Koshovyi, Jyrki Heinämäki, Alina Shpychak, Andres Meos, Niklas Sandler Topelius, Ain Raal

**Affiliations:** 1 Institute of Pharmacy, Faculty of Medicine, University of Tartu, 50144 Tartu, Estonia; oleh.koshovyi@ut.ee (O.K.); jyrki.heinamaki@ut.ee (J.H.); andres.meos@ut.ee (A.M.); 2 Department of Pharmacognosy and Nutriciology, The National University of Pharmacy, 61002 Kharkiv, Ukraine; shpichakalina@gmail.com; 3 CurifyLabs Oy, 00180 Helsinki, Finland; niklas.sandler@curifylabs.com

**Keywords:** 3D printing, semisolid extrusion, polyethylene oxide, printing gel ink, flavonoid, rutin

## Abstract

**Background/Objectives.** Flavonoids are a vast class of phenolic substances. To date, approximately 6000 plant-origin flavonoids have been discovered, with many of them being used in drug therapy. Therapeutic flavonoids are commonly formulated to conventional “one-size-fits-all” dosage forms, such as conventional tablets or hard capsules. However, the current trends in pharmacy and medicine are centred on personalised drug therapy and drug delivery systems (DDSs). Therefore, 3D printing is an interesting technique for designing and preparing novel personalised pharmaceuticals for flavonoids. The aim of the present study was to develop aqueous polyethylene oxide (PEO) gel inks loaded with rutin for semisolid extrusion (SSE) 3D printing. **Methods.** Rutin (a model substance for therapeutic flavonoids), Tween 80, PEO (MW approx. 900,000), ethanol, and purified water were used in PEO gels at different proportions. The viscosity and homogeneity of the gels were determined. The rutin–PEO gels were printed with a bench-top Hyrel 3D printer into lattices and discs, and their weight and effective surface area were investigated. **Results.** The key SSE 3D-printing process parameters were established and verified. The results showed the compatibility of rutin as a model flavonoid and PEO as a carrier polymer. The rutin content (%) and content uniformity of the 3D-printed preparations were assayed by UV spectrophotometry and high-performance liquid chromatography (HPLC). **Conclusions.** The most feasible aqueous PEO gel ink formulation for SSE 3D printing contained rutin 100 mg/mL and Tween 80 50 mg/mL in a 12% aqueous PEO gel. The 3D-printed dosage forms are intended for the oral administration of flavonoids.

## 1. Introduction

Flavonoids are phenolic substances that are abundant in plants and represent more than 6000 compounds [1,2]. Due to their chemical structure, flavonoids have significant therapeutic properties, including antioxidant, antibacterial, antiviral, and anti-inflammatory effects [3]. Flavonoid-based natural and semi-synthetic drugs are widely represented in the pharmaceutical market for the treatment of, for example, venous insufficiency (Diosmin, Troxerutin, and Hesperidin), gastric ulcers and gastritis (Eupatilin), and liver disorders (Silibinin) [4].

Rutin (3,3′,4′,5,7-pentahydroxyflavone-3-rhamnoglucoside), also known as quercetin-3-*O*-rutinoside and vitamin P, is one of the most common and wide-spread flavonoids [5]. Rutin is also an important nutrient component of plant-based food. Rutin was initially isolated from the common plant Rue (*Ruta graveolens* L.), from which it also received its name [3]. So far, it has been reported that more than 70 plant species contain rutin, including buckwheat (*Fagopyrum esculentum* Moench), Japanese pagoda tree (*Sophora japonica* L.), American elderberry (*Sambucus canadensis* L.), apricot (*Prunus armeniaca* L.), orange (*Citrus sinensis* L. Osbeck), white mulberry (*Morus alba* L.), rhubarb (*Rheum rhabarbarum* L.), etc. [5,6,7]. Buckwheat is considered one of the most profitable sources of rutin [8]. The quantitative content of the compound can reach up to 18.67 mg/g (dry weight) [9].

Rutin is an odourless and yellowish-greenish pigment in the form of needle crystals and is almost insoluble in water [3,10]. Due to its low solubility and low oral bioavailability, it has limitations in its use as a therapeutic agent [11,12]. To enhance the oral bioavailability of flavonoids, numerous formulation and delivery strategies have been introduced in the state-of-the-art literature, including structural transformation (glycosylation and prodrugs), nanotechnology-based carriers, crystals, micelles, carrier complexes, microspheres, self-microemulsifying drug delivery systems (DDSs), and self-nanoemulsifying DDSs [12,13,14].

Numerous scientific papers have reported that rutin possesses a wide range of pharmacological activities [3,6,15,16]. Rutin has been shown to have a strong concentration-dependent antioxidant effect in various in vitro systems and the ability to inhibit lipid peroxidation. Moreover, rutin has been shown to enhance the antioxidant status in the liver, kidneys, and brain of diabetic rats [6,17,18]. Rutin is also widely used in capillary fragility treatment, vascular sealing, and bleeding prevention, and as an antioxidant and anti-inflammatory agent in combination with ascorbic acid [10,19]. Many studies on rutin formulations have demonstrated significant antimicrobial activity against different strains of bacteria, including *Pseudomonas aeruginosa* and *Klebsiella pneumoniae* [6,17,20]. It has also been reported that rutin is an effective antiviral agent, which has shown affinity against the parainfluenza-3 virus, avian influenza virus, HSV-1, and HSV-2 [21]. In addition, rutin is obviously a potent inhibitor of COVID-19 infection treatment [21]. Recently, the anti-proliferative and anti-apoptotic activity of rutin was studied in an in vitro model with rutin-loaded liquid crystalline nanoparticles using a human lung epithelial carcinoma cell line [11]. The 50 mg/kg rutin dose decreased glucose levels by increasing insulin secretion in hyperglycaemic rats [5].

Today, virtually all flavonoid-containing commercial pharmaceuticals are in conventional “one-size-fits-all” oral solid dosage forms, such as tablets or hard capsules. Sometimes, however, such a traditional formulation approach is not appropriate, and a customised drug therapy and DDSs are needed. Therefore, 3D printing technologies are interesting and promising techniques for preparing novel types of personalised dosage forms for flavonoids [22,23]. By using a science-based material selection, formulation development, and computer-assisted design (CAD), modern 3D printing technologies could enable preparing next-generation customised oral DDSs for therapeutic flavonoids [23].

Semisolid extrusion (SSE) 3D printing is a printing technique widely used in pharmaceutical and biomedical applications [24]. SSE 3D printing exploits the sequential deposition of gel (or paste) layers to form the DDS or tissue engineering scaffold with the desired size and shape [24]. The present printing technique has proven efficient in delivering a precise and accurate personalised drug dosage and in preparing, for example, combinatorial DDSs with customised drug release properties [25]. Therefore, it holds considerable promise and potential in drug delivery applications. However, implementing SSE 3D printing technology in clinical practice requires new regulations and proper quality control approaches [26].

Viidik et al. [27] identified and optimised the critical material and process parameters of SSE 3D printing using aqueous polyethylene oxide (PEO) gels as a printing ink. More recently, PEO was successfully used as a printing gel ink former in the SSE 3D printing of novel DDSs of rosmarinic acid [28] and some plant extracts, such as eucalypt [29,30], chamomile [31], and cranberry [32] extracts. To our best knowledge, no research works have been published on the 3D printing of pure flavonoid substances using PEO as a printing gel former. Based on the abovementioned studies, PEO could be a feasible printing gel ink former for the SSE 3D printing of flavonoids, such as rutin.

The aim of the present study was to develop rutin-loaded aqueous PEO gel inks as model compounds of flavonoids for SSE 3D printing and to investigate the physicochemical and pharmaceutical properties of the corresponding solid DDSs intended for the oral administration of flavonoids. The physical appearance, homogeneity, viscosity, and SSE 3D printability of the rutin-loaded PEO gel inks were studied. The rutin content and in vitro disintegration of SSE 3D-printed oral DDSs were investigated.

## 2. Materials and Methods

### 2.1. Preparation of the PEO Gels

To present PEO gel inks, rutin (Nanjing NutriHerb BioTech Co, Nanjing, China, purity 95%), Tween 80 (Ferak Berlin GmbH, Berlin, Germany), PEO (MW approx. 900,000, Sigma-Aldrich, St. Louis, MO, USA), ethanol (Peenviinavabrik, Moe, Estonia), and purified water were used at different proportions. The 12% aqueous PEO gel was used as a semisolid ink base for the SSE 3D printing of rutin. First, 12% aqueous PEO gels were prepared, and then the mixture of rutin (0.5 g, 1.0 g, and 1.5 g in 1 mL of ethanol) with Tween 80 (at a rutin–Tween 80 ratio of 1:1, 1:2, or 1:3) was loaded in the PEO gel, homogenised, and kept at an ambient room temperature (22 ± 2 °C) for at least the subsequent 12–14 h [27].

### 2.2. Characterisation of the PEO Gels

The viscosity of PEO gels was studied with a Physica MCR 101 rheometer (Anton Paar, Graz, Austria) using cone–plate geometry at 22 ± 2 °C. A rotational shear test at 0.060 1/s was used [29] to measure the gel viscosity. The gel homogeneity and structure were determined with an optical light microscope (Magtex-T Dual Illum., Medline Scientific, Rotherham, UK) equipped with a digital camera (Industrial Digital Camera UCMOS09000KPB (9.0 MP 1/2.4″ APTNA CMOS sensor) [27,28].

### 2.3. SSE 3D Printing

The rutin–PEO gel inks were printed using a bench-top SSE 3D printing system (System 30 M, Hyrel 3D, Norcross, GA, USA) with a printing head consisting of a steel syringe connected to a blunt needle (Gauge, 21G). The printing plate temperature was kept at 30 °C. The printing head speed (rate) was 0.5 mm/s, and the printing speed was controlled with the software of an SSE 3D printer (Repetrel, Rev3.083_K, Hyrel 3D, Norcross, GA, USA). The model lattices were composed of a total of eight printed layers, and the round-shaped scaffolds consisted of five layers, which were generated with Autodesk 3ds Max Design 2017 software (Autodesk Inc., San Rafael, CA, USA) [28,29]. Round-shaped special scaffolds (20 mm in diameter) were designed using FreeCAD software (version 0.19/release date 2021). The dimensions (size) of square-shaped lattices were 30 × 30 × 0.5 mm. To verify the printability of the gel inks, the weight and area of the 3D-printed lattices were determined. The surface area of the 3D-printed lattices was compared with the theoretical lattice area (324 mm^2^) [29,33]. The 3D-printed scaffolds were weighed with an analytical scale (Scaltec SBC 33, Scaltec, Göttingen, Germany) and photographed with ImageJ (National Institute of Health, Bethesda, MD, USA) image analysis software (version 1.51k). The 3D-printed preparations were dried on a heated printing plate at 30 °C for one hour to remove the residual water. After drying, the printed scaffolds were gently removed from the surface of the printing plate using a special blade.

### 2.4. Disintegration Test In Vitro

The pilot in vitro disintegration test for the 3D-printed scaffolds was performed in a Petri dish filled with a small amount of purified water at room temperature (22 ± 2 °C) [28,29]. The goal of this pilot disintegration test was to verify if the 3D-printed scaffolds were rapidly disintegrating preparations or not. After this, we also conducted the established in vitro disintegration test described in the European Pharmacopoeia (Ph.Eur.), sub-monograph 2.9.1. “Disintegration of tablets and capsules” (01/2022:20901) [34]. The disintegration test (“test A”) was carried out in a Sotax DT3 CH-4008 apparatus (Sotax AG, Basel-Landschaft, Switzerland) without discs, using purified water (700 mL) as a disintegration medium at 37 ± 2 °C.

### 2.5. Dissolution Test In Vitro

The in vitro dissolution test was carried out in line with the method and protocol described in the European Pharmacopoeia, monograph 2.9.3: “Dissolution test for solid dosage forms” [35]. The in vitro dissolution of the rutin-loaded 3D-printed discs was investigated using a Sotax AT 7 Smart dissolution test apparatus (Sotax AG, Basel-Landschaft, Switzerland) equipped with an Ismatec IPC 8 ISM 931 peristaltic pump (Cole-Parmer Instrument Company LLC, Vernon Hills, IL, USA). Purified water (900 mL) was used as a dissolution medium at 37 ± 0.5 °C, and the paddle stirring rate was set at 50 rpm. The release of an active agent (rutin) was determined with a UV–Vis spectrophotometer (SPECORD 200 PLUS Dissolution, Analytik Jena GmbH+Co. KG, Jena, Germany).

### 2.6. Assay of Rutin Content by UV Spectrophotometry

The assay of rutin in the 3D-printed scaffolds was carried out by UV spectrophotometry after forming complexes with aluminium chloride [35]. The pre-weighed sample of a 3D-printed scaffold (100.00 mg) was dissolved in an aqueous isopropanol solution (50% *v*/*v*) *R* and analysed using a pharmacopeial method [33,35].

### 2.7. Assay of Rutin Content by HPLC

The quantitative content of rutin in the 3D-printed scaffolds was determined with a modified high-performance liquid chromatography (HPLC) method, according to the European Pharmacopoeia 11.5 “Rutoside trihydarate” monograph [34]. For the content analysis, Chromatograph Prominence Modular HPLC (Shimadzu, Japan) equipped with an online degassing unit (DGU-20ASR, 2 × Solvent Delivery Unit LC-20AD), a photo-diode array detector (SPD-M20A), an autosampler (Nexera X2 SIL-30AC), a Phenomenex Luna 5 µm C18(2) 100Å LC Column (250 × 4.6 mm), and a column oven (CTO-20AC) was used. LabSolutions Version 5.97 SP1 (c) 2019 (Shimadzu Corporation, Kyoto, Japan) was used to control the process. Rutin (PhytoLab, Vestenbergsgreuth, Germany, Catalogue no 89270, 50 mg, 99.0%, Batch 125820573) was used as a reference standard. The correlation between the peak area and the concentration was verified.

The pre-weighed sample of a 3D-printed scaffold (200.0 mg) was dissolved in an aqueous isopropanol solution (50% *v*/*v*) R and filled with the same solvent until 100.0 mL was reached. A total of 1.0 mL of the solution was filtered into a chromatography vial through a 0.45 µm nylon syringe filter. The analysis used 50 mg of rutin standard (Thermo Scientific, Waltham, MA, USA) dissolved in 100.0 mL aqueous ethanol (50% *v*/*v*) *R* as a standard solution. Phosphoric acid (1%), acetonitrile (25%), and water (74%) were used as the mobile phase. The flow rate was 1.0 mL/min. Spectrophotometric detection was carried out at the analytical wavelength of 330 nm. The injected volume was 10 µL, and the run time was 18 min.

### 2.8. Statistical Analysis

The statistical properties of random variables with *n*-dimensional normal distribution are given by their correlation matrices, which can be calculated from the original matrices. The statistical data assessment is reported as mean ± SEM. Statistical analysis was performed with a Welch *t*-test using MS Excel (Microsoft Excel 2016, version 16.0, Microsoft Corporation, Redmond, WA, USA). *p* values less than 0.05 indicate statistical significance [36].

## 3. Results

In the preliminary tests, we printed rutin-loaded 12% aqueous PEO gels without any additional excipients. Rutin is poorly soluble in water [6], so when its ethanolic solution was added to the aqueous PEO gel (without surfactants), the crystallisation/sedimentation of rutin occurred immediately. The crystals of rutin were clearly visible on the walls of the vessel. In such cases, it would be impossible to ensure the uniform dosing of the active ingredient, and it is, therefore, not necessary to confirm this phenomenon microscopically. Thus, the results were not satisfactory, since the homogeneity of the gels was quite poor, and rutin was partially oxidised during the 3D-printing process, which was manifested in brownish-coloured areas in the 3D-printed scaffolds. Since rutin is an antioxidant and phenolic compound, it readily undergoes redox reactions [6]. Therefore, we did not continue in this research line, concluding that this approach does not ensure a good printing quality for the final preparations.

Consequently, and following the reasons mentioned above, eumulgin or Tween 80 was added as a surface-active agent at a small concentration in the PEO printing gel ink to improve the homogeneity and printability of the gels. The inclusion of eumulgin in the PEO gel ink resulted in the 3D-printed scaffolds, which were readily removable from the printing plate surface; however, it was sometimes even impossible to terminate the SSE 3D-printing process. Next, we used Tween 80 at different concentrations (1, 3, and 5% *w*/*w*), finding that the most feasible concentrations of Tween 80 in the aqueous PEO gel inks were 3% and 5% (*w*/*w*). The corresponding PEO gel inks were homogeneous and showed great printability without any printing interruptions or flaws. Therefore, the present Tween-80-containing PEO gel ink was chosen for the next experimental rutin-loaded gel ink samples.

Table 1 shows the compositions of the Tween-80-containing rutin-loaded aqueous PEO gel inks, which were prepared and used for the SSE 3D printing experiments. To prepare such PEO gel inks, rutin was first dissolved in a small amount of ethanol, and Tween 80 was then added to this solution while gently stirring for 15 min. Next, the present mixture was added in driblets in an aqueous PEO gel ink and mixed gradually until a homogeneous gel was formed. Tween 80, a non-ionic surface-active agent, improved the homogeneity of the rutin-loaded aqueous PEO gels and prevented the precipitation and oxidation of rutin.

Table 2 shows the viscosity of the experimental rutin-loaded PEO gel inks. The viscosity of the gel inks was relatively high, but, on the other hand, was suitable for SSE 3D printing. Decreasing the amount of rutin from 1.00 g to 0.50 g appeared to increase the viscosity of the Tween-80-containing PEO gels. Statistically significant differences in the viscosity (*p* ≤ 0.01) were found between the PEO printing gels loaded with 1.00 g and 0.50 g of rutin.

**Figure 1:**
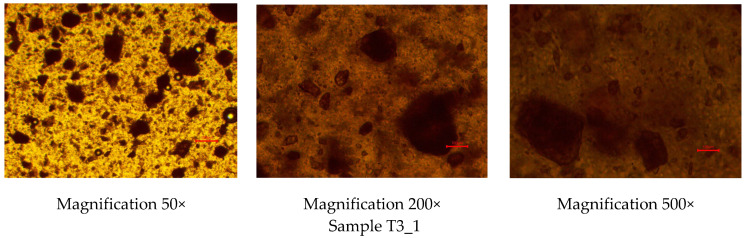

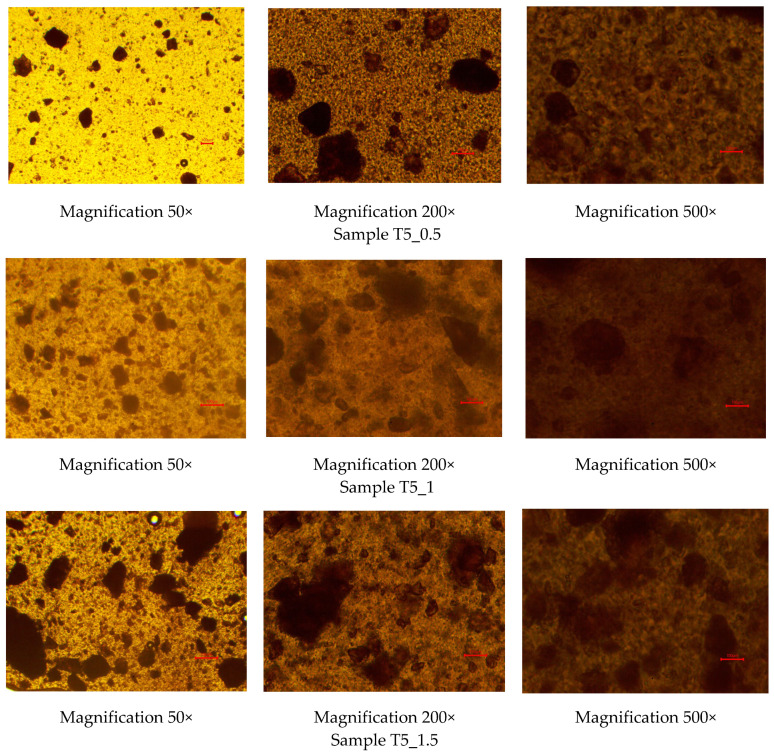
Optical light microscopy images of the rutin-loaded polyethylene oxide (PEO) printing gels. Magnification 50×, 200×, and 500×. Red scale is equal to 100 µm.

To investigate the homogeneity of the rutin-loaded PEO gel inks, the gels were studied under optical light microscopy immediately after preparation. Figure 1 shows the optical light microscopy images of the experimental PEO gel inks at three different magnifications (50×, 200×, and 500×). As seen in Figure 1, rutin was homogeneously dispersed in all Tween-80-containing aqueous PEO gels, thus suggesting the applicability of the present gels as printing inks in SSE 3D printing. No cluster formation was observed in the gel inks. The PEO gel inks containing rutin were yellow to brownish in colour.

**Figure 2:**
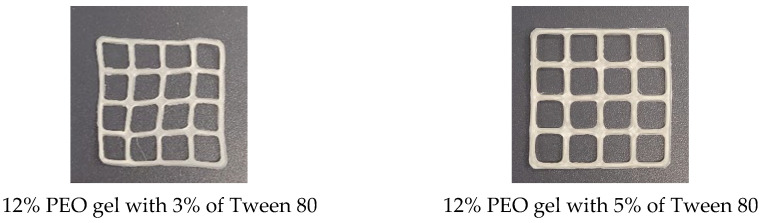

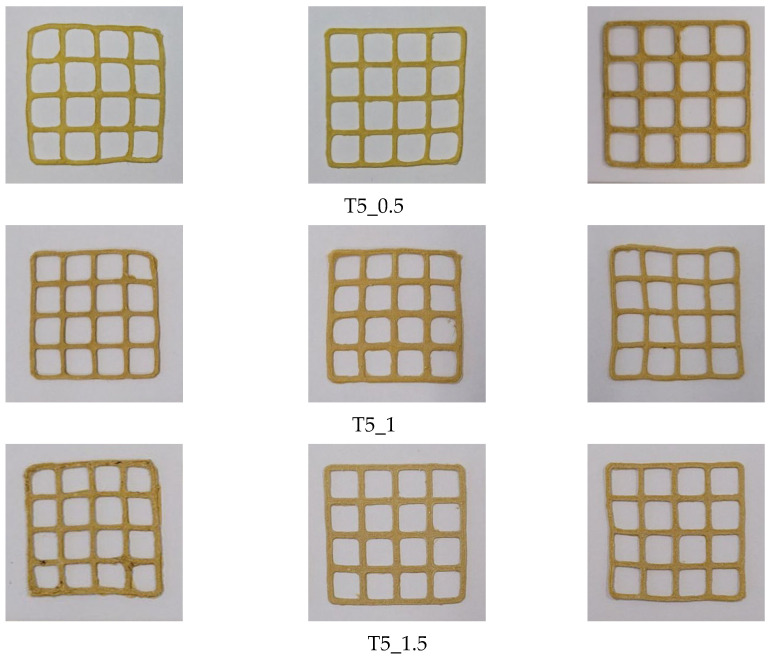
The semisolid extrusion (SSE) 3D-printed lattices prepared using polyethylene oxide (PEO) printing gels loaded with rutin and Tween 80 as an active agent and surface-active agent, respectively. Reference is also made to Table 1.

Figure 2 shows the physical appearance of the SSE 3D-printed lattices prepared using the rutin-loaded PEO gels. The two upper photographs present the reference 3D-printed lattices prepared using a 12% PEO gel ink containing Tween 80 as a surface-active agent, at 3% or 5% (*w*/*w*), without rutin. The photographs in the three lower rows of Figure 2 show the physical appearance of the rutin-loaded SSE 3D-printed lattices (three parallel printings) (reference is also made to Table 1). As seen in Figure 2, all three PEO gel inks loaded with rutin at three different concentrations showed very good printability. The lattices containing rutin were brownish to yellow in colour.

The surface area and weight of the SSE 3D-printed lattices of rutin are summarised in Table 3. The weight and surface area variation in the 3D-printed lattices were less than 12% and 10%, respectively.

We also used SSE 3D printing to prepare the round-shaped DDSs and scaffolds of rutin intended for oral administration (Table 4). The present scaffolds were uniform in colour, size, weight, and physical appearance. The weight variation in the 3D-printed lattices was less than 5%.

The content of rutin (%) in the SSE 3D-printed scaffolds was studied by means of UV spectrophotometry and HPLC, and the results are shown in Table 5.

As seen in Table 5, the rutin content in the SSE 3D-printed scaffolds was close to the theoretically calculated content value of the present flavonoid in the aqueous PEO gels, and the content uniformity was very good. The rutin assay results were also not dependent on the analytical method used. The rutin contents determined by UV spectrophotometry and HPLC were comparable with one another in terms of the theoretical content value and results.

**Figure 3:**
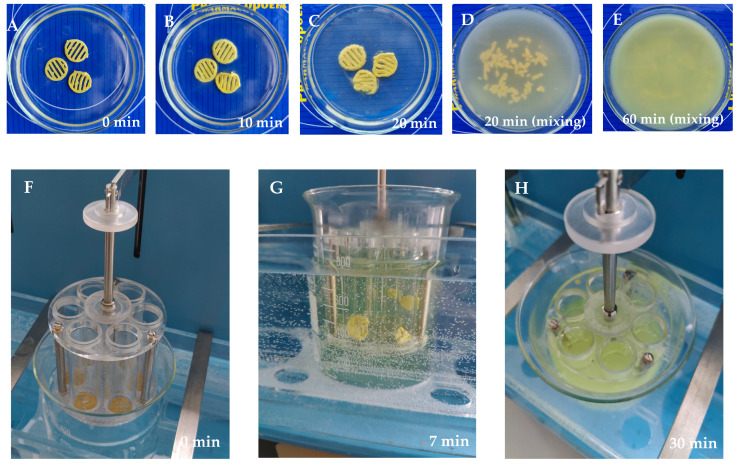
Disintegration of the semisolid extrusion (SSE) rutin-loaded 3D-printed polyethylene oxide (PEO) scaffolds in purified water at room temperature at 22 ± 2 °C (pilot test): (**A**) 0 min; (**B**) 10 min; (**C**) 20 min (without mixing); (**D**) 20 min (with gentle mixing); and (**E**) 60 min (with gentle mixing) (*n* = 3). Disintegration test according to the European Pharmacopoeia in purified water at 37 ± 0.5 °C: (**F**) 0 min; (**G**) 7 min; and (**H**) 30 min (*n* = 6).

Figure 3 shows the fate of the 3D-printed preparations in the in vitro disintegration test (a pilot test and an established Ph.Eur. disintegration test). Figure 3A–E shows that the 3D-printed scaffolds disintegrated completely within 30–60 min (a pilot test). Figure 3F–H shows that all 3D-printed preparations with different rutin concentrations (T5_0.5, T5_1, and T5_1.5) clearly deformed within 7 min, and the preparations completely disintegrated within 20–35 min. The 3D-printed preparations with the lowest rutin load (T5_0.5, 18.5% of rutin) disappeared from the tubes within 19–21 min, and the corresponding time periods for the 3D-printed preparations with the intermediate rutin load (T5_1, 30.1% of rutin) and the highest rutin load (T5_1.5, 41.1% of rutin) were 24–26 min and 32–35 min, respectively.

**Figure 4:**
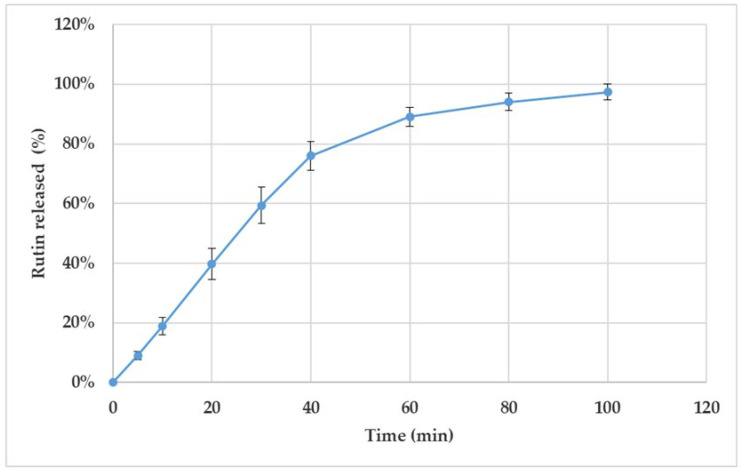
Dissolution of the semisolid extrusion (SSE) 3D-printed polyethylene oxide (PEO) discs loaded with rutin (T5_1). The content of rutin was 30.1% in the discs. The in vitro dissolution test was performed using purified water at 37 ± 0.5 °C as a dissolution medium (*n* = 4). The nonstandard number of parallel samples used in the test was considered as justified due to the uniform dissolution behaviour of the present 3D-printed discs.

Figure 4 shows the release behaviour of rutin from the 3D-printed discs (T5_1) in vitro. According to the European Pharmacopoeia monograph 2.9.3. “Dissolution test for solid dosage forms” [35], the established time period for performing the dissolution test in vitro is 60 min. In our study, however, the preliminary results showed that 81–89% of rutin was released from the 3D-printed scaffolds within 60 min. Therefore, we continued the dissolution test up to 100 min. As seen in Figure 4, approximately 88–103% of rutin was released within 80 min, and virtually all of the active agent load was released within 100 min.

## 4. Discussion

In the present study, we formulated and evaluated aqueous PEO gel inks loaded with plant-origin rutin for SSE 3D printing. The PEO gel inks loaded with rutin and Tween 80 as a surface-active agent were found to be homogeneous, viscous gels that are yellow in colour. The addition of rutin (1.0 g) to the 12% aqueous PEO gels (10 g) without any surface-active agent leads to the impaired homogeneity of the gels and to colour changes in the final 3D-printed scaffolds due to the oxidation of rutin. Rutin has low solubility in water [3,6], which caused immediate precipitation when its ethanolic solution was mixed with the 12% aqueous PEO gel (without surfactants). The crystals of rutin were observed on the walls of the vessel. In such cases, it would be impossible to ensure the uniform dosing of the active ingredient, and we, therefore, did not confirm this phenomenon microscopically.

Since rutin is an antioxidant and phenolic compound, it is prone to redox reactions [3,6]. In our preliminary studies, we found that brown-coloured zones were formed in the 3D-printed scaffolds when rutin was applied without any excipients. This was obviously due to the oxidation of rutin. Therefore, to avoid such unexpected phenomena, surface-active agents, such as Emulgin and Tween 80, were used in the printing gel inks. When Emulgin was added to the PEO gel inks, we found that the 3D-printed scaffolds readily (too quickly) peeled off from the printing plate, which, in some cases, prevented the successful completion of the 3D printing. Therefore, Tween 80 was selected as a surface-active agent for the PEO gel inks [37]. The use of Tween 80 enabled a more homogeneous distribution of rutin in the PEO gels, as well as preventing the oxidation of rutin and providing very good 3D printing results. By using Tween 80, the brown-coloured zones were not formed (distinguishable) in the 3D-printed scaffolds. Moreover, the HPLC analysis (Figure 3) did not reveal any additional peaks, thus indicating that no structural changes in rutin took place under these conditions. Among the three concentration levels of Tween 80 studied, using the two highest concentrations of Tween 80 (3% and 5% *w*/*w*) [38] resulted in good stability and printability of the PEO gel. The PEO gel inks with the lowest Tween 80 concentration level (1% *w*/*w*) were not homogeneous or stable.

When preparing the rutin-loaded PEO printing gel inks, the order of mixing and adding ingredients was crucial. If the procedure was violated, it was impossible to obtain homogeneous gels. First, rutin was dissolved in a minimal volume of ethanol. This liquid volume needs to be considered and adjusted with the volume of water used in preparing the PEO gel inks for 3D printing. Then, the pre-calculated amount of Tween 80 was added in driblets to this solution and thoroughly mixed. PEO gel inks should be prepared according to the standard procedure, considering the volume of ethanol used to dissolve the rutin [27]. Finally, the ethanolic rutin solution with Tween 80 was gradually added to the PEO gel while continuously stirring. This procedure ensured the preparation of homogeneous gels, which was confirmed by visual inspection and by means of optical light microscopy (Figure 1). Vibrational spectroscopic (FTIR and Raman) microscopy techniques, however, would be the methods of choice for more profound homogeneity evaluations of the present printing gels.

Our hypothesis is that, based on their physicochemical properties, surface-active agents (Tween 80 and Emulgin) [38,39,40,41] can help to prevent the oxidation of rutin during SSE 3D printing through several mechanisms: (1) the formation of a protective layer, (2) the stabilisation of rutin in a solution, (3) the reduction in free radicals, (4) by regulating pH, and (5) by preventing shear-induced oxidation. The above-mentioned mechanisms jointly support the chemical stability and integrity of rutin during 3D-printing processes and, consequently, prevent the oxidation of rutin. The mechanical stress of SSE 3D printing may foster oxidation by generating heat or shear forces. Surface-active agents can reduce surface tension and improve the flow properties of printing gels [42], thus minimising the mechanical stress and reducing the chance of oxidation.

The PEO gels loaded with rutin at all three concentrations and prepared as described in the previous paragraph showed good printability in the SSE 3D-printing process (Figure 2, Table 3). We used three different rutin concentrations in the printing gels (0.5, 1.0, and 1.5 g in 10 mL of the gel). In the 3D-printed round scaffolds, the rutin dose was 17 mg, 50 mg, or 67 mg based on the concentration of rutin in the printing gel. Using PEO gels with a rutin concentration of 100 mg/mL (T5_1) led to the highest quality of printed scaffolds based on visual inspection and surface area analysis [27,30]. With the present printing gel, it is possible to print rutin-loaded preparations of any size, shape, and dose. By increasing the number of printed layers in these preparations, the dose of rutin can be increased accordingly.

The results of the disintegration test in vitro showed that the SSE 3D-printed PEO scaffolds completely lost their shape and disintegrated within 15–20 min in purified water at room temperature, 22 ± 2 °C (Figure 3A–E). We also conducted the established in vitro disintegration test (“test A”) described in the European Pharmacopoeia (Ph.Eur.), sub-monograph 2.9.1. “Disintegration of tablets and capsules” (01/2022:20901) [35]. We showed that all 3D-printed preparations (*n* = 6) disintegrated completely within 20–35 min in this additional in vitro disintegration test (Ph.Eur.), thus showing a rapid disintegration behaviour (Figure 3F–H). As expected, the disintegration of 3D-printed preparations was dependent on the amount (load) of rutin, since rutin is poorly soluble in water [6]. Increasing the amount (load) of rutin also increased the disintegration time of the preparations. Higher contents of hydrophobic drugs (rutin) (Table 5) impaired the wetting of the printed drug preparations, thus hindering the disintegration of the preparations (the amount of a surface-active agent, Tween-80, was 5% in the printing gel, Table 1). According to the European Pharmacopoeia [35], the specified time period for the disintegration of oral tablets and hard capsules is 15 min and 30 min, respectively.

As seen in Figure 4, the amount of rutin released from the 3D-printed discs (*n* = 4) was uniform over the entire studied time period. Moreover, with all four drug preparations, the rutin load was completely released (and dissolved) within approximately 2 h. The variation in the dissolution behaviour could be due to the slight differences in the geometric shapes of the printed and cut drug preparations. During the first 40 min, a linear release profile as a function of time was obtained for rutin. This linear part of the dissolution curve indicated that rutin release from the 3D-printed discs followed the constant release rate up to 40 min.

Our results suggest that the present SSE 3D-printed preparations have potential for oral administration. The disintegration of the present 3D-printed preparations can be accelerated by the inclusion of a disintegrant (or “super-disintegrant”) in the formulation. The main mechanisms of the commonly used pharmaceutical disintegrants and “super-disintegrants” are swelling, capillary action (water imbibition), or gas generation in the constructs [43,44]. Pharmaceutical “super-disintegrants,” such as sodium starch glycolate [45] and crosslinked sodium carboxymethyl cellulose, typically act at a very low concentration (2–5%), which is an advantage from the printed formulation point of view [38,46]. Further research work is needed to find the most effective and compatible disintegrant for the present 3D-printed preparations loaded with poorly water-soluble rutin.

Today, the European Pharmacopoeia [35] does not have any specific monograph or disintegration/dissolution test methods for 3D-printed oral solid dosage forms. We found that the European Pharmacopoeia dissolution test method (described in sub-monograph 2.9.3) is also feasible for testing 3D-printed drug preparations intended for oral administration. For the assay of rutin, we used the validated analytical methods described in the European Pharmacopoeia, with only a slight modification [34,35]. Both UV spectrophotometry and the HPLC method showed good reproducibility in the content analysis of rutin. The assay results of rutin were in line with the theoretical rutin content and were comparable with each other. Even though the present analytical methods are established pharmacopoeia methods, the further development of these methods for the assay of rutin [47,48] is important, particularly regarding sample preparation. In our current study, we demonstrated that these two analytical methods described in the European Pharmacopoeia are feasible for the content analysis of SSE rutin-loaded 3D-printed preparations.

The novelty of our research work lies in the successful formulation of an aqueous-based printing gel ink for SSE 3D printing using PEO as a gel former and rutin flavonoids as an active agent. The effects of two surface-active agents on the 3D printing behaviour of such aqueous-based gels were verified. To the best of our knowledge, no research works have been published on the application of SSE 3D printing for the oral preparation of plant-based flavonoids. Therefore, the present research work is a pioneer study in this field and could open new formulation strategies for the 3D printing of plant-origin flavonoids. There were some earlier attempts to print hydroxycinnamic acids, such as rosmarinic acid [28], and plant extracts [29,30,31,32], however, in these studies, the flavonoid content in the 3D-printed constructs was very small (only up to 7%). Since flavonoids possess significant antioxidant properties [1,2,6], their concentration can affect the performance of 3D printing and the quality of the final printed preparations. In the present work, we changed both the strategy for adding the emulsifier to a PEO printing gel and the type of emulsifier. In previous studies, the emulsifying agents were added in proportion to the active substance content [28,29,30,31,32]. We also showed that the inclusion of Tween 80 at a concentration of 5% enhances the distribution and stability of rutin in the PEO gel base. This means that we were able to reduce the concentration of the emulsifier threefold. While the optimal PEO printing gel of rosmarinic acid contained 150 mg/mL of emulsifier [28], our current study showed that the rutin-loaded PEO gels require only 50 mg/mL of Tween 80.

Since rutin is an established and well-known representative of plant-origin flavonoids, the SSE 3D-printed formulations (i.e., printing gel inks) developed in our study are most likely also feasible in the SSE 3D printing of other plant-origin flavonoids with potential therapeutic value and can be implemented into pharmaceutical practice.

## 5. Conclusions

Novel aqueous PEO gel ink compositions were developed for the pharmaceutical SSE 3D printing of plant-origin rutin. The present 3D-printed formulations and customised drug preparations could also be used for the oral administration of other plant-origin flavonoids with potential therapeutic value. The PEO gel inks loaded with rutin and Tween 80 (as a surface-active agent) showed good printability at printing head speed (rate) levels of 0.5 mm/s. The most feasible aqueous PEO gel formulation for the SSE 3D printing of rutin was composed of rutin at 100 mg/mL and Tween 80 as a surface-active agent at 50 mg/mL (dissolved in a 12% aqueous PEO gel). Further studies are needed to reveal potential physicochemical incompatibilities between the material components in the present PEO gel inks and to verify the in vitro dissolution properties and storage stability of the SSE 3D-printed DDSs of rutin.

## Figures and Tables

**Table 1:** Composition of the rutin-loaded polyethylene oxide (PEO) printing gels.

Exp.	Rutin, g	Tween 80, g	PEO, g	Ethanol, mL	Water, mL
T3_1	1.00	0.30	1.20	1.00	9.00
T5_0.5	0.50	0.50	1.20	1.00	9.00
T5_1	1.00	0.50	1.20	1.00	9.00
T5_1.5	1.50	0.50	1.20	2.00	8.00

**Table 2:** The viscosity of the rutin-loaded polyethylene oxide (PEO) printing gels (*n* = 3).

Sample	Viscosity, cP (Speed 0.03 RPM, Shear Rate 0.060 1/s, Temperature 22 ± 2 °C, *n* = 3)
T3_1	219,867 ± 27,380
T5_0.5	250,867 ± 13,169 *
T5_1	223,367 ± 14,712
T5_1.5	226,567 ± 9845

* Statistically significant difference between T3_1 and T5_0.5 (*p* ≤ 0.01) in a Welch *t*-test.

**Table 3:** Weight and surface area of the semisolid extrusion (SSE) 3D-printed polyethylene oxide (PEO)-based lattices loaded with rutin and Tween 80 (*n* = 3).

Sample	Weight, mg	Area (S), mm^2^	S_practical/_S_theoretical_
T5_0.5	117.7 ± 1.3	336.3 ± 28.5	1.04
T5_1	163.0 ± 18.0	339.9 ± 20.8	1.05
T5_1.5	184.2 ± 16.4	389.2 ± 24.4	1.20

**Table 4:** The semisolid extrusion (SSE) 3D-printed discs were prepared using polyethylene oxide (PEO) printing gels loaded with rutin and Tween 80 as an active agent and surface-active agent, respectively. Reference is also made to Table 1.

Sample	Weight, mg	Photographs
T5_0.5	90.2 ± 4.2	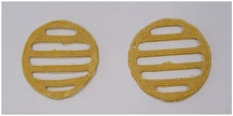
T5_1	168.2 ± 8.4	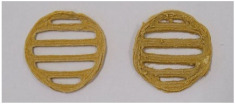
T5_1.5	165.7 ± 7.8	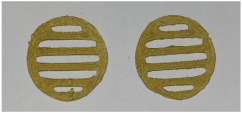

**Table 5:** Assay of rutin in the semisolid extrusion (SSE) 3D-printed scaffolds using UV spectrophotometric and high-performance liquid chromatography (HPLC) methods.

Sample	Content of Rutin, % (*n* = 3)		
	Theoretical *	UV Spectrophotometry	HPLC
T5_0.5	22.72 *	17.23 ± 0.23	18.5
T5_1	36.79 *	31.21 ± 0.36	30.1
T5_1.5	46.42 *	42.35 ± 0.49	41.1

Note. *—the moisture content in the starting substance was not taken into account in the calculations.

## Data Availability

The data supporting the results of this study can be obtained from the corresponding authors upon reasonable request.
